# Novel Inflammatory Biomarkers for Autism Spectrum Disorder Detected by Plasma Olink Proteomics

**DOI:** 10.3390/children12020210

**Published:** 2025-02-11

**Authors:** Tiying Lv, Mingbang Wang, Ling Kui, Jun Wu, Yang Xiao

**Affiliations:** 1 Department of Integrative Medicine, Jinshazhou Hospital of Guangzhou University of Chinese Medicine, Guangzhou 510623, China; 20222410020@stu.gzucm.edu.cn; 2 Department of Neonatology, Longgang District Maternity & Child Healthcare Hospital of Shenzhen City (Longgang Maternity and Child Institute of Shantou University Medical College), Shenzhen 518172, China; mingbang_wang@szu.edu.cn; 3 Microbiome Therapy Center, Department of Experiment & Research, South China Hospital, Medical School, Shenzhen University, Shenzhen 518111, China; 4 Shenzhen Qianhai Shekou Free Trade Zone Hospital, Shenzhen 518067, China; kuiling2008@163.com (L.K.); wujun197306@126.com (J.W.)

**Keywords:** autism spectrum disorder, inflammation, biomarker, IL-17C, Olink

## Abstract

**Background:** Research evidence has recently shown an association between autism spectrum disorder (ASD) and inflammation. For example, the expression of inflammatory cytokines is abnormal in children with ASD, and maternal inflammation can lead to ASD-like behavior in offspring. These studies suggest that inflammation plays an important role in the occurrence and development of ASD. Inflammatory cytokines may, therefore, be potential biomarkers for ASD. In the present study, we sought to systematically identify inflammatory biomarkers of children with ASD. **Methods:** We used Olink proteomics to comprehensively examine differentially expressed inflammation-related proteins in 60 children with ASD and 28 children with typical development (TD). We validated our findings using published data. **Results:** A total of 18 inflammation-related proteins were differentially expressed between the ASD and TD groups. Compared with the TD group, the expression of all differentially expressed proteins was up-regulated in the ASD group. Furthermore, eight differentially expressed proteins showed good diagnostic efficacy, as delineated by area under the curve (AUC) values of > 0.7. To our knowledge, this is the first time that up-regulated interleukin-17C (IL-17C), chemokine ligand (CCL)-19, and CCL20 have been detected in the plasma of children with ASD (with AUC of 0.839, 0.763, and 0.756, respectively). We also found that there was a negative correlation between inflammatory cytokines and SRS scores. **Conclusions:** Multiple inflammatory markers were increased in children with ASD. IL-17C, CCL19, and CCL20 exhibit potential as biomarker candidates for ASD. Elevated expression levels of cytokines may enhance social ability in ASD.

## 1. Introduction

Autism spectrum disorder (ASD) is a neurodevelopmental disorder characterized by impairments in social communication, repetitive behaviors, and restricted interests that appear in early childhood [1]. ASD has high disability and heritability rates and is incurable, magnifying the financial burdens on individuals, families, and society. Early diagnosis is key to improving prognosis. However, the high clinical heterogeneity of ASD makes diagnosis difficult despite clearly defined diagnostic criteria in both the Diagnostic and Statistical Manual of Mental Disorders, Fifth Edition (DSM-5) and the International Classification of Diseases, 11th Revision (ICD11). Current diagnosis mainly relies on the subjective judgment of doctors, yet for children whose symptoms are less clear-cut, the management and treatment of the disease will be delayed. Therefore, the development of effective diagnostic biomarkers for ASD is urgently needed.

The incidence of ASD has been increasing in recent years. However, its underlying mechanisms remain poorly understood. The interaction of genetics and early environmental factors plays a key role in the etiology of ASD [2], with inflammation being a significant environmental risk factor. Extensive evidence suggests that inflammation contributes to the onset and progression of various neurological disorders, including Alzheimer’s disease, Parkinson’s disease, multiple sclerosis, and ASD. Moreover, ASD has been associated with abnormal inflammatory responses in both the peripheral and central nervous systems [3,4,5,6]. For instance, children with ASD exhibit increased expression levels of cytokines such as tumor necrosis factor (TNF), interleukin (IL)-8, and C-X-C motif chemokine ligand (CXCL)-6 compared to typically developing (TD) children [7,8]. Additionally, studies on maternal immune activation (MIA) models have demonstrated that inflammation can induce ASD-like behaviors [9]. Notably, these ASD-like behaviors in MIA models can be reversed by blocking cytokines [10], further suggesting that immune dysfunction and inflammatory responses play critical roles in ASD pathogenesis. Inflammation can infiltrate the brain via the blood-brain barrier, triggering neuroinflammation [11] and subsequently leading to abnormal behaviors. There is evidence that microglia and astrocytes in the brain and cerebrospinal fluid are significantly activated in children with ASD, accompanied by up-regulation of pro-inflammatory factors [12]. As the primary innate immune cells in the central nervous system (CNS), microglia and astrocytes respond to stimuli by producing pro-inflammatory cytokines and chemokines (such as TNF, IL-1β, IL-16, CCL2, and IL-18) to eliminate pathological factors. They serve as key regulators of CNS inflammation [13]. Although it is not clear whether neuroinflammation is a direct pathogenic factor of ASD, it appears to be an important cofactor in disease progression. Anti-inflammatory and immunosuppressive drugs may represent a novel strategy for the treatment of ASD [14,15,16,17]. Drugs such as prednisolone, celecoxib, and minocycline have been shown to improve irritability, hyperactivity, and lethargy in ASD and have demonstrated a partial therapeutic effect on core symptoms (e.g., stereotypical behavior) in a small sample study [18]. Further, cytokine levels are associated with abnormal behavior in ASD [19,20]. These studies suggest that inflammatory cytokines may serve as potential biomarkers for ASD diagnosis and severity assessment. Thus, we propose that inflammation-related proteins could be potential biomarkers for ASD.

Omics technology is an important tool for discovering biomarkers and understanding the pathophysiological mechanisms of complex diseases. Protein biomarkers have always been central to disease prediction, diagnosis, and defense, reflecting both abnormalities in upstream DNA or RNA molecules and the effects of external stimuli. Traditional proteomic techniques have various limitations in terms of throughput, sensitivity, and practical clinical ability [21]. The most common method, mass spectrometry, is expensive and limited by the abundance effect, whereby the presence of high-abundance molecules masks the detection of low-abundance molecules. Recently, the new proteomic platform, Olink, has been widely recognized because its proximity extension assay (PEA) has good repeatability and stability. A PEA uses antibody pairs conjugated to complementary oligonucleotides, which are amplified, allowing highly sensitive and specific, multiplexed measurements with only a small amount of blood [22]. Unlike mass spectrometry, the amplification steps in PEA allow less abundant proteins, including cytokines or chemokines, to be better detected. This ensures accuracy and reliability of the assay [23]. Therefore, in this study, we used Olink proteomics to systematically evaluate the level of inflammatory cytokines in children with ASD and identify potential inflammatory biomarkers for ASD.

## 2. Materials and Methods

### 2.1. Participants

The protocol for this study was approved by the ethics committee of Shenzhen Qianhai Shekou Free Trade Zone Hospital (approval number: 2023k-004). Plasma samples were collected from 60 children with ASD and 28 children with TD, aged 2–12 years, from Shenzhen Qianhai Shekou Free Trade Zone Hospital (Shenzhen, China) between November 2023 and February 2024. The inclusion criteria for TD children were as follows: healthy children with no family history of neurological or psychiatric disorders, both male and female, aged 2 to 12 years. The inclusion criteria for children with ASD included a diagnosis of ASD according to the DSM-5 criteria, Childhood Autism Rating Scale (CARS) scores exceeding 30, both sexes, and aged 2 to 12 years. The exclusion criteria were as follows: children with other neurodevelopmental disorders that do not meet the core ASD symptoms (social communication deficits and restricted, repetitive patterns of behavior, interests, or activities), including intellectual disability, communication disorders, developmental coordination disorder, stereotypic movement disorder, and global developmental delay; children diagnosed with schizophrenia, deafness, and organic nervous system diseases; children with diseases affecting the heart, liver, or kidneys; children with inflammatory or infectious diseases or those on ongoing medication during the study period. Children’s symptoms were assessed using the childhood autism rating scale (CARS) score. Clinical manifestations were determined using the autism behavior checklist (ABC), social responsiveness scale (SRS), and repetitive behavior scale-revised (RBS-R).

### 2.2. Plasma Sample Collection

In total, 88 plasma samples were collected from the participants. Approximately 2 mL of peripheral venous blood was collected from each participant into ethylene diamine tetraacetic acid tubes. Then, plasma was extracted by centrifugation at 4 °C (1500× *g* for 10 min) and frozen at −80 °C for future use.

### 2.3. Inflammation-Related Biomarkers Analysis

Plasma samples from ASD (n = 60) and TD (n = 28) groups were analyzed using the Olink inflammation panel, based on the highly sensitive and specific PEA technology, which enabled 92 inflammation-related proteins to be analyzed simultaneously. Each sample was tested once, with a quality control assessment conducted afterward. A PEA combines the specificity of antibody technology with a DNA amplification step. It achieves good repeatability and stability by binding two antibodies to a single protein, bringing their conjugated, complementary DNA strands into proximity, and amplifying and detecting the resulting double-stranded DNA. The final assay readout was presented in normalized protein expression values, which were log2-transformed.

### 2.4. Bioinformatics Analysis

All statistical analyses were performed using R 4.4.2 [24] (R Foundation for Statistical Computing, Vienna, Austria. URL https://www.R-project.org/, accessed on 12 November 2024) and IBM SPSS Statistics 26(IBM Corp. Released 2017, Armonk, NY, USA: IBM Corp.). Olink data were input into R using the “OlinkAnalyze” package. Orthogonal Partial Least Squares Discriminant Analysis (OPLS-DA) is a widely applied technique in omics research, particularly suited for multivariate studies where variables exhibit interdependence. In this study, OPLS-DA was conducted using MetaboAnalyst 6.0 (https://www.metaboanalyst.ca/, accessed on 26 January 2025) to minimize noise and facilitate dimensionality reduction. Features with a Variable Importance in Projection (VIP) score exceeding 1.0 were deemed significant contributors to the model. Gene ontology (GO) and Kyoto Encyclopedia of Genes and Genomes (KEGG) enrichment analyses were performed using the “ggplot2 3.5.1” package. The diagnostic performance of differentially expressed proteins (DEPs) was determined by receiver operating characteristic (ROC) curves generated using the “ROCR 1.0-11” package. A higher area under the curve (AUC) value reflects better diagnostic efficiency. Protein–protein interaction (PPI) networks of DEPs were constructed using the STRING database (https://string-db.org/, accessed on 12 November 2024) and visualized using Cytoscape 3.7.2 [25] (https://cytoscape.org/, accessed on 21 July 2023).

### 2.5. Validation

A literature search was conducted on ASD and proteomics using the Embase (https://www.embase.com/, accessed on 25 November 2024) and PubMed (https://pubmed.ncbi.nlm.nih.gov/, accessed on 25 November 2024) databases. To avoid additional confounding factors, a previous study using plasma samples with Olink proteomics was included for validation [26]. The DEPs identified in the present study were used for logistic regression of published data. The AUC values are shown in the legend in the lower right corner.

### 2.6. Statistical Analysis

Data are expressed as mean ± standard deviation or median (Q1, Q3), as appropriate. IBM SPSS Statistics 25 and R software (version 4.4.2) were used for statistical analyses. Values of *p* < 0.05 were considered statistically significant.

## 3. Results

### 3.1. Participant Demographics

The cohort included a total of 88 participants (n = 60 ASD and 28 TD). The majority of children with ASD were male (78.33%), with a male-to-female ratio of 3.6:1, consistent with a previous epidemiological report [27]. Table 1 shows the clinical characteristics of the participants. There were no significant differences in age and sex ratio between the two groups (*p* > 0.05).

### 3.2. Olink Inflammation-Related Biomarker Analysis

**Figure 1:**
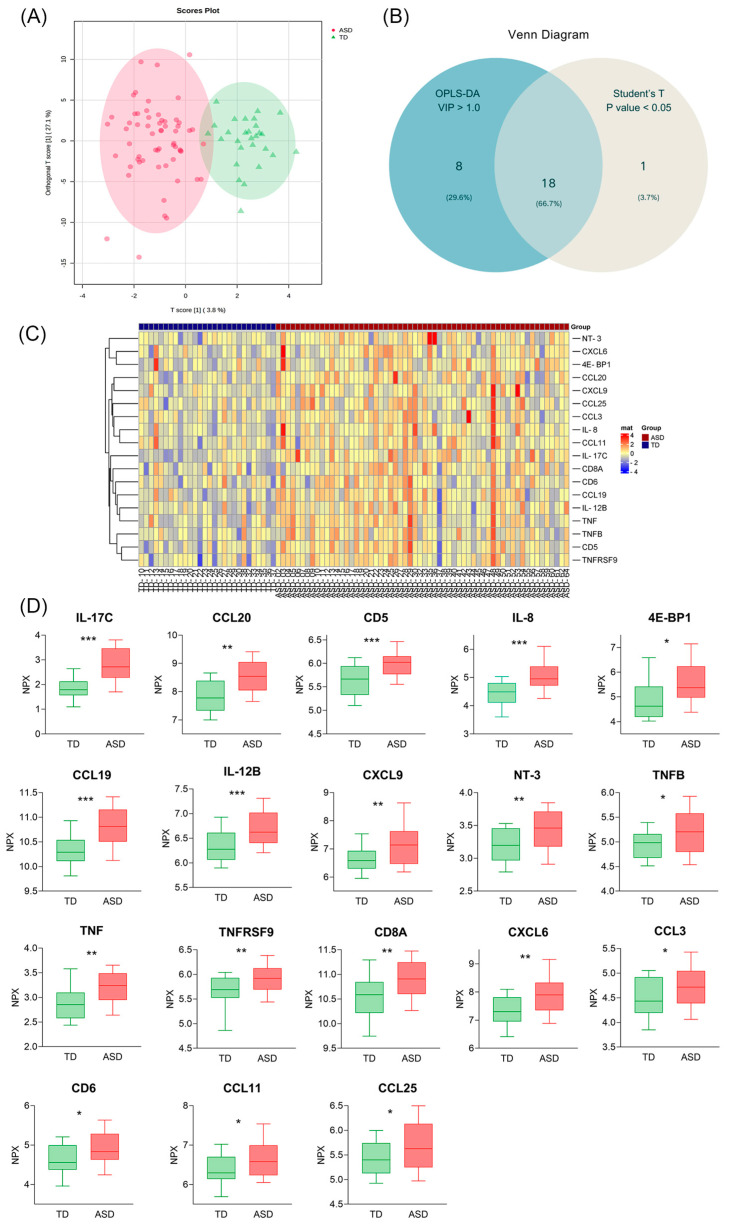
Differentially expressed inflammation-related proteins in ASD and TD groups. (**A**) Score plot of the orthogonal partial least squares regressions discriminant analysis (OPLS-DA) model delineating the separation of autism spectrum disorder (ASD) and typical development (TD) groups based on protein expressions (NPX). (**B**) Venn diagram showing proteins with a Variable Importance in Projection (VIP) score > 1.0 (calculated via OPLS-DA) and *p*-value < 0.05 (calculated via Student’s *t*-test). (**C**) Heat maps of 18 differentially expressed proteins (DEPs). (**D**) Box plots of these 18 DEPs. (* *p* < 0.05, ** *p* < 0.01, *** *p* < 0.001).

The expression levels of 92 inflammation-related proteins were compared in the ASD and TD groups using Olink analysis. Detailed information on the 92 proteins is provided in Table S1. In the ASD group, most inflammatory proteins were increased, consistent with previous studies [28]. Using OPLS-DA, we distinguished between the ASD and TD groups (Figure 1A) and identified 26 inflammatory proteins with a VIP score greater than 1.0. A comprehensive list is provided in Table S2. Differentially expressed proteins (DEPs) were further screened based on VIP scores (VIP > 1.0) and *t*-test *p*-values (*p* < 0.05), as detailed in Table 2 and Figure 1B. Eighteen DEPs were identified, with a detailed list available in Table 2 and Figure 1C. Among these, IL-17C had the highest VIP score (2.37451). To our knowledge, this is the first report of up-regulated IL-17C, chemokine ligand (CCL) 19, and CCL20 in the plasma of children with ASD.

### 3.3. Analysis of Differentially Expressed Inflammation-Related Biomarkers

**Figure 2:**
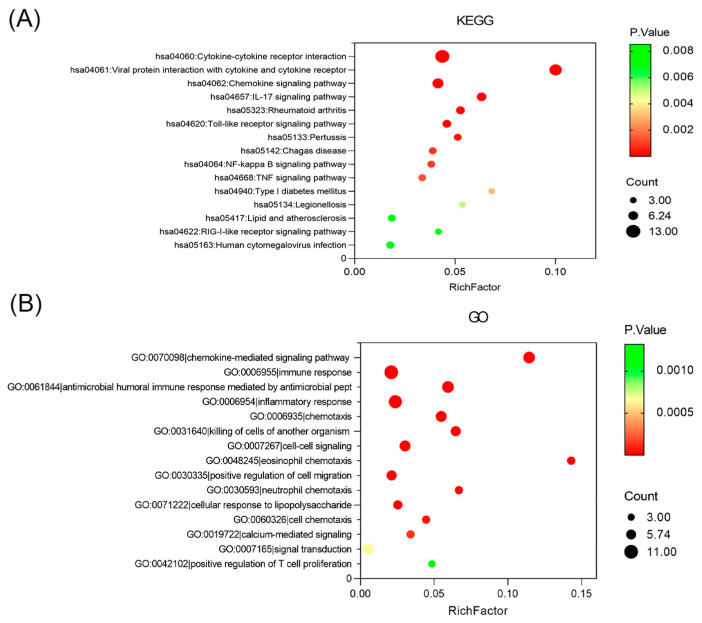
Enrichment analyses of DEPs by GO and KEGG. (**A**) KEGG pathway enrichment analysis based on all annotated proteins (**B**) GO enrichment analysis based on all annotated proteins. KEGG, Kyoto Encyclopedia of Genes and Genomes; GO, Gene Ontology.

To further investigate the potential function of the DEPs, we performed GO and KEGG enrichment analyses. On a background of all annotated proteins, several pathways were involved, such as the cytokine–cytokine receptor interaction, IL-17 signaling pathway, toll-like receptor (TLR) signaling pathway, and nuclear factor-κB (NF-κB) signaling pathway, consistent with previous studies [10,29] (Figure 2A). Additionally, GO enrichment analysis revealed that the DEPs were primarily associated with chemokine-mediated signaling, immune responses, and inflammatory processes (Figure 2B).

**Figure 3:**
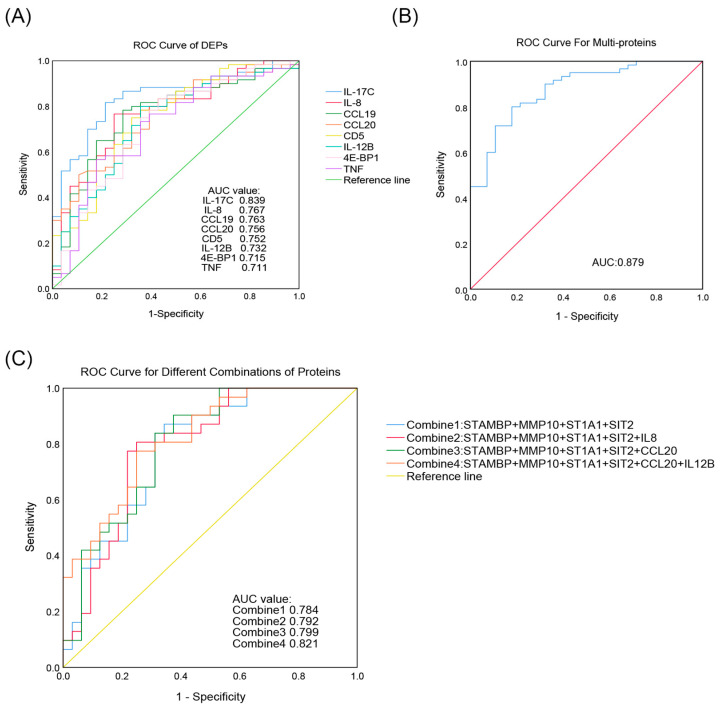
ROC curves of individual and multi-proteins. (**A**) ROC curves of DEPs with AUC > 0.7. (**B**) ROC curve for multi-proteins with AUC > 0.7. (**C**) ROC curves for different combinations of proteins from published data. ROC, receiver operating characteristic; AUC, area under the curve.

ROC curves for the 18 DEPs were plotted based on sensitivity and specificity, and the AUCs were individually computed. This showed that 8 of 18 DEPs had an AUC of > 0.7 (95% confidence interval [95%CI]) and were of potential diagnostic significance (Figure 3A). These were IL-17C (0.839 [0.754–0.924]), IL-8 (0.767 [0.662–0.872]), CCL19 (0.763 [0.655–0.871]), CCL20 (0.756 [0.652–0.859]), cluster of differentiation (CD) 5 (0.752 [0.642–0.863]), IL-12B (0.732 [0.618–0.846]), eukaryotic translation initiation factor 4E-binding protein 1 (4E-BP1) (0.715 [0.5955–0.836]), and TNF (0.711 [0.592–0.831]). Of these, IL-17C had the highest AUC value and was deemed a better diagnostic classifier than the other seven DEPs. A logistic regression model was calculated for the ROC curves of these eight DEPs. A classifier composed of these DEPs was higher than a single IL-17C, with an increase in AUC from 0.839 (0.754–0.924) to 0.879 (0.805–0.902) (Figure 3B). The AUC values for all DEPs are provided in Table S3.

Due to the small sample size of the present study and the high heterogeneity of ASD, our classifier model may be overfitted and difficult to apply to other cohorts. However, we believe that some DEPs may have a role in improving the diagnostic efficiency of the model. Combine1 was the original regression model based on published data, with an AUC value of 0.784 (0.671–0.898). Adding our DEPs, such as IL-8 (Combine2) and CCL20 (Combine3), to the Combine1 model, AUC values increased from 0.784 to 0.792 (0.679–0.906) and 0.799 (0.689–0.909), respectively. CCL20 combined with IL-12B (Combine4) contributed to better diagnostic performance, increasing the AUC value to 0.821 (0.720–0.921).

### 3.4. Protein–Protein Interaction Analysis

**Figure 4:**
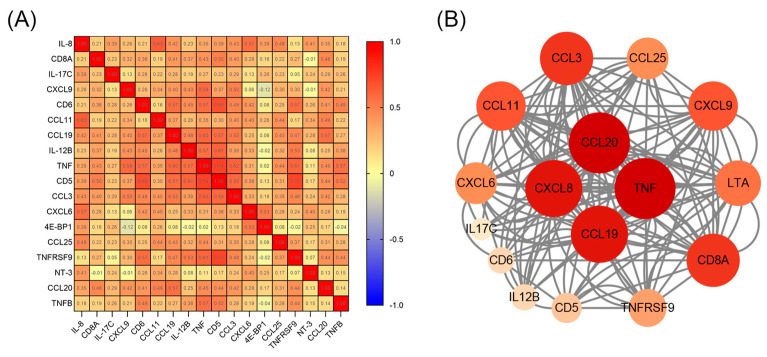
Correlation and interaction between DEPs. (**A**) Heat map showing correlation between DEPs in children with ASD. Red, positively related; blue, negatively related; and yellow, unrelated. Correlation coefficients were calculated using Spearman’s correlation test and are marked in the corresponding grids. (**B**) Protein–protein interaction network analysis of DEPs.

Considering the likelihood of a cascade of inflammation, we conducted a correlation analysis between the 18 inflammatory proteins (Figure 4A). Unsurprisingly, we found many associations between inflammatory proteins, among which CD5 and TNF were most significantly positively correlated (r = 0.739, *p* < 0.01). To explore interactions between the 18 DEPs, we annotated them using the STRING database and mapped the PPI network using Cytoscape. This showed that TNF, CCL20, CXCL8, and CCL19 had higher degree scores in the PPI network, suggesting they may play an important role in ASD (Figure 4B).

**Figure 5:**
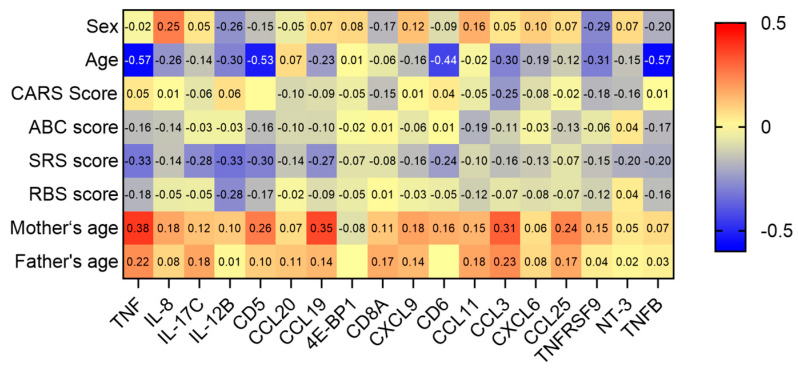
Heat map showing the correlation between DEPs and clinical characteristics. Red, positively related; blue, negatively related; and yellow, unrelated. Correlation coefficients were calculated using Spearman’s correlation test and are marked in the corresponding grids.

Subsequently, we examined the correlation between the 18 DEPs and the clinical characteristics of children with ASD. This showed that IL-17C, CCL19, IL-12B, IL-13, TNF, and CD5 levels were negatively correlated with SRS scores in children with ASD, while CD6 and CCL20 were associated with social awareness from SRS, and tumor necrosis factor β (TNFB) was associated with autistic mannerisms from SRS. Furthermore, IL-12B was associated with RBS-R scores in children with ASD, while CCL20 and TNF were associated with self-injurious and sameness from RBS-R, respectively (Figure 5). Finally, CCL19, TNF, CD5, and CCL3 were positively correlated with maternal age but were independent of the father’s age. The results of the complete Spearman correlation analyses are provided in Table S4.

## 4. Discussion

In this study, we compared the plasma expression levels of 92 inflammation-related proteins between ASD and TD participants using an Olink inflammation panel. We identified 18 inflammation-related proteins with significantly different expressions between the two groups. Our findings suggest that children with ASD exhibit abnormal inflammatory responses, as reflected in their protein expression levels, compared to children with TD. These identified DEPs were associated with clinical symptoms and age, and they hold potential as inflammatory biomarkers for ASD. Following KEGG and GO enrichment analyses, we identified enrichment of these DEPs in several inflammatory pathways, including the NF-κB signaling pathway, IL-17 signaling pathway, and TLR signaling pathway. Thus, inflammation may be involved in the progression of ASD through several pathways. Our study provides further supporting evidence for immune dysregulation in children with ASD, contributing to a deeper understanding of the physiological and pathological mechanisms underlying ASD and aiding in the identification of potential candidate biomarkers associated with the disorder.

**Figure 6:**
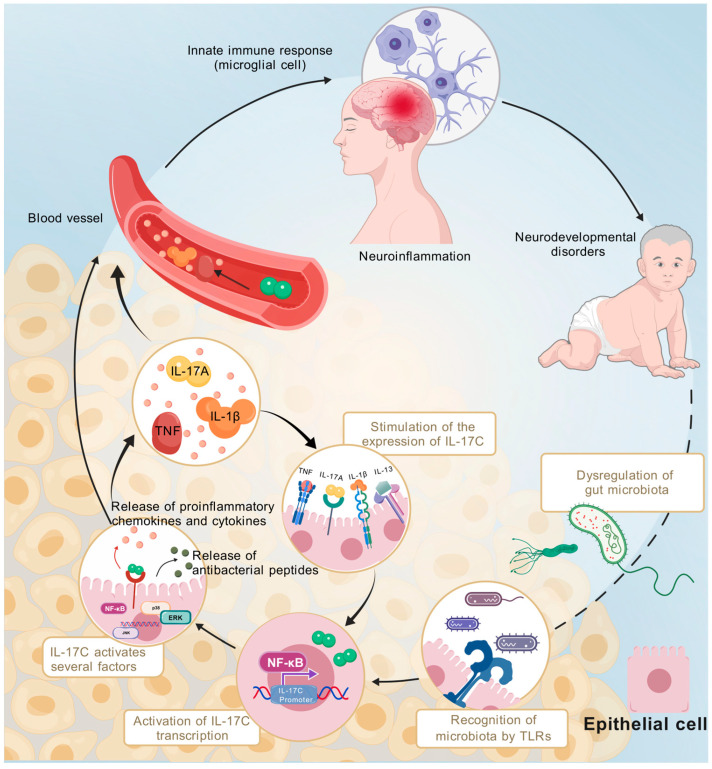
The potential mechanism linking IL-17C in the pathogenesis of ASD. Gut microbiomes play a vital role in human health. During intestinal gut dysbacteriosis, harmful bacteria are increased and recognized by TLRs on the surface of colon epithelial cells. This increases the expression of IL-17C through the NF-κB pathway. At the same time, IL-17C interacts with IL-17RE to release pro-inflammatory chemokines and cytokines, such as TNF, IL-1β, and IL-17A, thereby activating multiple pathways. These products further stimulate IL-17C expression. Eventually, IL-17C and pro-inflammatory chemokines and cytokines enter the brain through blood circulation, activating the innate immune response of microglia. This can ultimately affect the neurological development of children, resulting in the occurrence of ASD. The figure was created with BioGDP.com [47].

Among the DEPs, eight had an AUC > 0.7, indicating good classification potential. Five of the proteins (TNF, CD5, IL-8, IL12B, and 4E-BP1) were abnormally elevated in children with ASD [30,31,32,33,34], consistent with previous studies. To our knowledge, CCL20, CCL19, and IL-17C are reported here for the first time as potential biomarkers for ASD. CCL20 and CCL19 are CC chemokines that attract lymphocytes and mild neutrophils. By binding and activating the chemokine receptors, CC chemokine receptor (CCR) 6 and CCR7, they can induce the mobilization of intracellular calcium ions, which mediates the effects of cancer, various autoimmune diseases, and antimicrobial responses. Both of these proteins play key roles in the formation and maintenance of chronic nerve inflammation, and affect the proliferation and survival of neuronal cells [35,36,37]. They are well-known biomarkers for other neurological disorders, such as schizophrenia and stroke. IL-17C is up-regulated in several neurological diseases, such as Alzheimer’s disease [38], bipolar disorder [39], and stroke [40], but to the best of our knowledge, has not been previously reported in ASD. In our study, IL-17C had the highest AUC value among all DEPs, suggesting that IL-17C may be an inflammatory cytokine with the potential to develop into a biomarker for ASD. Although IL-17C belongs to the IL-17 family, it is mainly secreted by epithelial cells (rather than immune cells), specifically respiratory epithelial cells and colon epithelial cells. IL-17C exerts its effects primarily by binding to the IL-17 receptor E (IL-17RE) [41], which is mainly distributed in the gastrointestinal tract. In host defense, gut microbiota dysbiosis increases expression levels of IL-17C in the intestine through the TLR signaling pathway [42]. After binding with IL-17RE on the surface of epithelial cells, IL17C, via an actin adaptor protein (Act1), initiates downstream signaling (e.g., through NF-κB, ERK, JNK, and p38) to induce the expression of pro-inflammatory chemokines, cytokines, antimicrobial peptides, and other host defense molecules [43]. Excessive IL-17C and inflammatory cytokines can also cause neuroinflammation and brain damage by activating the innate immune response of microglia. Microglia play an important role in maintaining normal brain function [44]. The use of anti-IL-17C neutralizing antibodies can alleviate polystyrene nanoplastic-induced neurotoxicity [45]. These studies suggest that IL-17C may play an important role in ASD through the gut–brain axis. In addition to the TLR signaling pathway, TNF and IL-1β can also induce the expression of IL-17C in epithelial cells [46] (Figure 6).

Enrichment analyses showed that DEPs were mainly enriched in cytokine–cytokine receptor interactions and TLR, NF-κB, and IL-17 signaling pathways, similar to previous studies [10,29]. TLRs are a key player in the innate immune system, the body’s first line of defense against infection and tissue damage. TLRs can activate the IκB kinase complex and lead to activation of mitogen-activated protein kinase (MAPK) and transcription of NF-κB, which controls the expression of many inflammatory cytokine genes. IL-17, similar to TLRs, can trigger NF-κB and MAPK signaling pathways to induce downstream genes, thereby promoting the progression of autoimmune diseases, host defense, and tumor progression [48,49]. This plays an important role in immune regulation and homeostasis. Our PPI analysis showed that TNF, IL-8, CCL20, and CCL19 were closely related to each other and all correlated with SRS scores in children. TNF and IL-8, which are downstream factors of the NF-κB pathway, are common abnormal inflammatory cytokines of ASD. They are significantly elevated in the early stage of ASD and are considered potential diagnostic biomarkers for ASD [50,51]. CCL19 and CCL20 are sensitive to hypoxia, inflammation, and edema in the brain and can be up-regulated in the early stage of cerebrovascular disease; they are important indicators of stroke. Although interactions between these four proteins are still unclear, they may play important roles in the pathophysiology of ASD through inflammatory pathways such as viral protein interactions with cytokines and cytokine receptors, cytokine–cytokine receptor interactions, and the NF-κB signaling pathway.

We conducted correlation analyses between DEPs and clinical characteristics. Of these DEPs, the age-related proteins were IL-8, CD6, IL-12B, TNF, CD5, CCL3, tumor necrosis factor receptor superfamily member (TNFRSF)9, and TNFB. Although these proteins demonstrated some classification potential in this study, the results should be viewed with caution until more generalizable results are obtained after detailed stratification of a large sample study. We also found that maternal age correlated with CCL19, TNF, CD5, and CCL3 in children, which is consistent with the results of previous studies [52,53]. However, no significant correlation was observed with paternal age, and the reason for this remains unclear [54]. Further research is necessary to investigate whether the observed inflammatory responses are primarily driven by genetic factors, environmental influences, or a combination of both [52,55]. Future studies should aim to elucidate the underlying biological and environmental mechanisms contributing to these associations.

Although inflammation and social behavior are not generally thought to be related, our findings suggest that inflammation may play an important role in social behavior. More surprisingly, there was a negative correlation between inflammatory cytokines and SRS scores, suggesting that elevated expression levels of inflammatory cytokines may enhance the social ability of children with ASD. This contradicts previous assumptions that increased inflammation leads to socially withdrawn behaviors. However, other studies have found that inflammation actually leads to increased socialization patterns and sensitivity to social rewards in some cases. For example, rhesus monkeys exposed to lipopolysaccharide showed a marked increase in social behavior, spending more time with their caged companions [56]. Consistent with these behavioral findings in animals, participants treated with endotoxins also show greater ventral striatum (VS) activity when viewing images of people they are close to compared with placebo [57]. VS is a reward-related region, and it has been shown to be associated with social connection. Moreover, the study indicated that the increase in pro-inflammatory cytokines in the endotoxin response was positively correlated with an increase in VS activity. These findings suggest that inflammation can lead to increased sensitivity to the reward of intimate others and also increased desire to be close to intimate others [58]. Previous studies suggest that this phenomenon may be related to social stress or the need for comfort during illness. However, these explanations do not seem to fully account for the observations in children with ASD. The association of inflammatory biomarkers with social function may help identify therapeutic interventions to prevent and treat social deficits. The hypothesis of an abnormal inflammatory response in ASD is supported by a considerable body of literature, but the association between inflammation and clinical features of ASD and the mechanism behind it are still unclear and need further research.

Due to the small study sample size, high heterogeneity of ASD, a cascade effect between inflammatory cytokines, and the doping of age factors, the regression model may be overfitted and difficult to apply directly to other cohorts. However, we found that some DEPs may have a role in improving diagnostic efficiency. Based on a model of published data [26], AUC increased from 0.784 to 0.821 after including CCL20 and IL-12B in the logistic regression model. Although the difference between multiple ROCs was not significant (*p* > 0.05), possibly due to the small validation cohort, it is still worthy of further exploration. The present study had several limitations. First, the number of participants in the study cohort was small due to difficulties in obtaining blood samples from children. Follow-up research is needed in larger patient cohorts. Second, the study was conducted at a single center, which may limit its generalizability to all children with ASD. The validation in multi-center studies is necessary. Third, our findings were derived from a single-omics analysis, and additional multi-omics approaches could provide deeper insights into the biological mechanisms involved. Despite these limitations, the present study has identified several plasma inflammatory proteins that were elevated in ASD. Moreover, while our results suggest that certain inflammatory proteins may serve as potential biomarkers for ASD, further research is needed to clarify their role in ASD pathogenesis and progression and to rigorously assess their potential as diagnostic biomarkers.

## Figures and Tables

**Table 1:** Participant demographics for the clinical samples subjected to Olink analysis.

Characteristics	ASD (n = 60)	TD (n = 28)	*p* Values ^1^
Sex			
Male	47	18	0.162 (Chi-square test)
Female	13	10	
Age (years), median (Q1,Q3)	5.00 (4.00,7.00)	6.50 (4.25,8.00)	0.074 (Mann-Whitney test)

ASD, autism spectrum disorder; TD, typical development; Q1, first quartile; Q3, third quartile. ^1^ *p* values were calculated using the Chi-square test (sex) and Mann–Whitney *U* test (age).

**Table 2:** Significantly different proteins between ASD and TD groups.

Proteins Symbol	Uniport	VIP ^1^	*p* Value ^2^
IL-17C	Q9P0M4	2.37451	0.0000
CCL20	P78556	1.85226	0.0000
CD5	P06127	1.90184	0.0000
IL-8	P10145	1.85046	0.0001
CCL19	Q99731	1.82337	0.0001
IL-12B	P29460	1.51174	0.0009
CXCL9	Q07325	1.27995	0.0016
NT-3	P20783	1.45186	0.0019
TNF	P01375	1.53776	0.0025
TNFRSF9	Q07011	1.54543	0.0034
CD8A	P01732	1.32687	0.0036
CXCL6	P80162	1.34411	0.0050
4E-BP1	Q13541	1.39000	0.0100
TNFB	P01374	1.28974	0.0117
CCL3	P10147	1.20120	0.0129
CCL25	O15444	1.12928	0.0131
CD6	P30203	1.2468	0.0131
CCL11	P51671	1.18062	0.0275

^1^ VIP was calculated via OPLS-DA; ^2^ *p* values were calculated via Student’s *t*-test.

## Data Availability

All data generated or analyzed during this study are included in this article and its Supplementary Materials; further inquiries can be directed to the corresponding author.

## Supplementary Materials

The following supporting information can be downloaded at https://www.mdpi.com/article/10.3390/children12020210/s1, Table S1: Information of 92 inflammation-related proteins in Olink analysis; Table S2: Inflammatory proteins with a VIP score greater than 1.0; Table S3: AUC (95% CI) for 19 DEPs comparing ASD to TD; Table S4: Spearman correlation analyses between DEPs and clinical characteristics; Data S1: Raw data of the Olink proteomics analysis.
