# Supplementary material for: Novel Inflammatory Biomarkers for Autism Spectrum Disorder Detected by Plasma Olink Proteomics
Source: Children (Basel). 2025 Feb 11;12(2):210. doi: 10.3390/children12020210 (PMC11853758; doi:10.3390/children12020210)
Supplement: Supplementary file 1 [file children-12-00210-s001.zip › Supplementary Material.pdf]

Supplementary Materials

Novel inflammatory biomarkers for autism spectrum disorders detected by plasma Olink proteomics

Table S1. Information of 92 inflammation-related proteins in Olink analysis.

Protein Symbol	Uniprot ID	Name
4E-BP1	Q13541	Eukaryotic translation initiation factor 4E-binding protein 1
ADA	P00813	Adenosine Deaminase
ARTN	Q5T4W7	Artemin
AXIN1	O15169	Axin-1
Beta-NGF	P01138	Beta-nerve growth factor
CASP-8	Q14790	Caspase-8
CCL11	P51671	Eotaxin
CCL19	Q99731	C-C motif chemokine 19
CCL20	P78556	C-C motif chemokine 20
CCL23	P55773	C-C motif chemokine 23
CCL25	O15444	C-C motif chemokine 25
CCL28	Q9NRJ3	C-C motif chemokine 28
CCL3	P10147	C-C motif chemokine 3
CCL4	P13236	C-C motif chemokine 4
CD244	Q9BZW8	Natural killer cell receptor 2B4

CD40	P25942	CD40L receptor
CD5	P06127	T-cell surface glycoprotein CD5
CD6	P30203	T cell surface glycoprotein CD6 isoform
CD8A	P01732	T-cell surface glycoprotein CD8 alpha chain
CDCP1	Q9H5V8	CUB domain-containing protein 1
CSF-1	P09603	Macrophage colony-stimulating factor 1
CST5	P28325	Cystatin D
CX3CL1	P78423	Fractalkine
CXCL1	P09341	C-X-C motif chemokine 1
CXCL10	P02778	C-X-C motif chemokine 10
CXCL11	O14625	C-X-C motif chemokine 11
CXCL5	P42830	C-X-C motif chemokine 5
CXCL6	P80162	C-X-C motif chemokine 6
CXCL9	Q07325	C-X-C motif chemokine 9
DNER	Q8NFT8	Delta and Notch-like epidermal growth factor-related receptor
EN-RAGE	P80511	Protein S100-A12
FGF-19	O95750	Fibroblast growth factor 19
FGF-21	Q9NSA1	Fibroblast growth factor 21
FGF-23	Q9GZV9	Fibroblast growth factor 23
FGF-5	P12034	Fibroblast growth factor 5

Flt3L	P49771	Fms-related tyrosine kinase 3 ligand
GDNF	P39905	Glial cell line-derived neurotrophic factor
HGF	P14210	Hepatocyte growth factor
IFN-gamma	P01579	Interferon gamma
IL-1 alpha	P01583	Interleukin-1 alpha
IL10	P22301	Interleukin-10
IL-10RA	Q13651	Interleukin-10 receptor subunit alpha
IL-10RB	Q08334	Interleukin-10 receptor subunit beta
IL-12B	P29460	Interleukin-12 subunit beta
IL-13	P35225	Interleukin-13
IL-15RA	Q13261	Interleukin-15 receptor subunit alpha
IL-17A	Q16552	Interleukin-17A
IL-17C	Q9P0M4	Interleukin-17C
IL-18	Q14116	Interleukin-18
IL-18R1	Q13478	Interleukin-18 receptor 1
IL-2	P60568	Interleukin-2
IL-20	Q9NYY1	Interleukin-20
IL-20RA	Q9UHF4	Interleukin-20 receptor subunit alpha
IL-22 RA1	Q8N6P7	Interleukin-22 receptor subunit alpha-1
IL-24	Q13007	Interleukin-24

IL-2RB	P14784	Interleukin-2 receptor subunit beta
IL-33	O95760	Interleukin-33
IL-4	P05112	Interleukin-4
IL5	P05113	Interleukin-5
IL6	P05231	Interleukin-6
IL-7	P13232	Interleukin-7
IL-8	P10145	Interleukin-8
LAP TGF-beta-1	P01137	Latency-associated peptide transforming growth factor beta-1
LIF	P15018	Leukemia inhibitory factor
LIF-R	P42702	Leukemia inhibitory factor receptor
MCP-1	P13500	Monocyte chemotactic protein 1
MCP-2	P80075	Monocyte chemotactic protein 2
MCP-3	P80098	Monocyte chemotactic protein 3
MCP-4	Q99616	Monocyte chemotactic protein 4
MMP-1	P03956	Matrix metalloproteinase-1
MMP-10	P09238	Matrix metalloproteinase-10
NRTN	Q99748	Neurturin
NT-3	P20783	Neurotrophin-3
OPG	O00300	Osteoprotegerin
OSM	P13725	Oncostatin-M

PD-L1	Q9NZQ7	Programmed cell death 1 ligand 1
SCF	P21583	Stem cell factor
SIRT2	Q8IXJ6	SIR2-like protein 2
SLAMF1	Q13291	Signaling lymphocytic activation molecule
ST1A1	P50225	Sulfotransferase 1A1
STAMBP	O95630	STAM-binding protein
TGF-alpha	P01135	Transforming growth factor alpha
TNF	P01375	Tumor necrosis factor
TNFB	P01374	TNF-beta
TNFRSF9	Q07011	Tumor necrosis factor receptor superfamily member 9
TNFSF14	O43557	Tumor necrosis factor ligand superfamily member 14
TRAIL	P50591	TNF-related apoptosis-inducing ligand
TRANCE	O14788	TNF-related activation-induced cytokine
TSLP	Q969D9	Thymic stromal lymphopoietin
TWEAK	O43508	Tumor necrosis factor (Ligand) superfamily, member 12
uPA	P00749	Urokinase-type plasminogen activator
VEGF-A	P15692	Vascular endothelial growth factor A

Table S2. inflammatory proteins with a VIP score greater than 1.0.

Protein	VIP[t]	VIP[ot]

IL-17C	3.19491145151941	0.395726640728751
CCL20	2.38095410749281	0.863578955923184
CD5	2.3417985312879	1.17349680090746
IL-8	2.29791456642921	1.09420713910071
CCL19	2.29261338367111	1.00377811617225
NT-3	1.91040159605083	0.508137893169528
TNFRSF9	1.83349689103309	1.10481792447081
IL-12B	1.78530884282116	1.09693178177687
TNF	1.77450739898486	1.19329137032171
CD8A	1.67065256385919	0.724080497973401
CXCL9	1.62440297813994	0.661873319392834
4E-BP1	1.60339202783554	1.07971291080268
CXCL6	1.44847849055208	1.20704277596494
IL33	1.39142713894122	0.787718490452709
CD6	1.34210617969356	1.12182693803764
TNFB	1.31962206806645	1.25295227399049
CCL3	1.3162058951254	1.04669233844624
CCL25	1.27223070701984	0.929207054731921
DNER	1.25884580277905	1.16037997941784
IL-15RA	1.20393854785305	1.19920597070044

CCL11	1.19504301302575	1.1630712150009
CX3CL1	1.19294998309087	0.992218876242549
IL-17A	1.16363082573351	0.890702132978557
Flt3L	1.07780130522266	0.690187050672882
uPA	1.02824340545491	0.870638461356943
OSM	1.00848904400907	1.13848085185319

VIP, Variable Importance in Projection

Table S3. AUC (95% CI) for 19 DEPs comparing ASD to TD.

Protein Symbol	AUC (95% CI)
IL-17C	0.839(0.754-0.924)
IL-8	0.767(0.662-0.872)
CCL19	0.763(0.655-0.871)
CCL20	0.756(0.652-0.859)
CD5	0.752(0.642-0.863)
IL-12B	0.732(0.618-0.846)
4E-BP1	0.715(0.595-0.836)
TNF	0.711(0.592-0.831)
CD8A	0.700(0.580-0.820)
NT-3	0.694(0.584-0.804)
CXCL9	0.690(0.576-0.804)

CXCL6	0.689(0.572-0.805)
TNFRSF9	0.688(0.574-0.802)
CD6	0.662(0.541-0.783)
CCL3	0.651(0.529-0.772)
CCL11	0.649(0.527-0.772)
TNFB	0.642(0.525-0.759)
CCL25	0.620(0.502-0.739)
IL-13	0.564(0.442-0.685)

AUC, the areas under the receiver operating characteristic curves; CI, confidence interval; DEP, differentially expressed protein; ASD, autism spectrum disorder; TD, typical development.
